# Anti-mitotic chemotherapeutics promote apoptosis through TL1A-activated death receptor 3 in cancer cells

**DOI:** 10.1038/s41422-018-0018-6

**Published:** 2018-03-01

**Authors:** Chen Qi, Xin Wang, Zhirong Shen, She Chen, Hong Yu, Noelle Williams, Gelin Wang

**Affiliations:** 1 School of Pharmaceutical Sciences, Tsinghua University, Beijing, 100084 China; 2 BeiGene, Beijing, 102206 China; 3 National Institute of Biological Sciences, Beijing, 102206 China; 4 Department of Biochemistry, University of Texas Southwestern Medical Center, Dallas, TX 75390-9152 USA

## Abstract

The commonly used antimitotic chemotherapeutic agents such as taxol and vinblastine arrest cell cycle progression by disrupting mitotic spindles, and cause cancer cells to undergo apoptosis through ‘mitotic catastrophe’. The molecular mechanisms by which these drugs induce apoptosis and their relevance to clinical efficacy are not known. Facilitated by a new spindle poison diazonamide, we found that apoptosis induced by these agents requires death receptor 3 (DR3). Mitotic arrest by these agents induces lysosome-dependent secretion of the DR3 ligand, TL1A. Engagement of TL1A with DR3 stimulates the formation of FADD-containing and caspase-8-containing death-inducing signaling complex (DISC), which subsequently activates apoptosis in cells that express DR3. Expression of DR3 and TL1A correlates with the apoptotic response of human tumor xenograft models and human cancer cell lines to antimitotic drugs, providing further evidence that these drugs kill cancer cells through the DR3/TL1A-mediated pathway. These results suggest that TL1A and DR3 may hold promise to be used as biomarkers for predicting clinical response to antimitotic therapeutics.

## Introduction

The most distinguishing hallmark of cancer is uncontrolled cell growth and division. Chemical and biological agents that antagonize these features are therefore most commonly used in the clinical treatment of cancer. Among those are tubulin-targeting agents such as taxanes and Vinca alkaloids that either stabilize microtubules or prevent microtubules from assembling. Since microtubules are important components of mitotic spindles, the disruption of microtubule dynamics by these drugs arrests cell division, thereby preventing cancer growth.^1–3^ Although being widely used in the clinic as a standard therapy for many human cancers and having demonstrated substantive therapeutic efficacy, anti-tubulin therapies have significant limitations. First, tubulin is ubiquitously utilized in both cancerous and normal cells. It is anticipated that tubulin-binding drugs display significant toxicities in normal tissue. Second, the antitumor activities of these drugs appear to have tissue specificities. For example, it is not known why anti-tubulin drugs are often effective against ovarian, mammary, lung and hematological cancers, but essentially ineffective against kidney, colon, or pancreas cancers.^4^ Even for the same type of cancer, patient response rates are varied and unpredictable, which might be due to the tumor metastasis. Some cellular determinants of sensitivity and resistance to these drugs clearly exist.

Diazonamide is a new class of marine natural products that show remarkable activity in inhibiting cancer cell growth when tested in a panel of 60 NIH cell lines.^5^ The pattern of the inhibition mirrors other tubulin destabilizing agents.^6,7^ Diazonamide itself is not a good tubulin binder and its precise mechanism of action remains to be determined although it has been shown to bind to ornithine amino transferase (OAT) with high affinity.^8^ The relevance of OAT and other diazonamide-interacting proteins to its antimitotic function remains unclear. However, it was found that diazonamide caused mitotic spindle dysfunction, which could primarily contribute to its killing effects on cancer cells and xenografted tumor.^7,8^ Diazonamide functions differently from other antimitotics when administered to eliminate xenograft tumors. It preserves the microtubule network in non-dividing cells and in primary neurons; does not cause any body-weight loss, any change in overall physical appearance, or any evidence of neutropenia; and functions as effectively as taxanes and vinca alkaloids. These demonstrate that diazonamide has a remarkably larger therapeutic window compared to taxanes and vinca alkaloids in rodents.^9,10^ The selective toxicity of diazonamide toward tumors and our access to the synthetic diazonamide derivatives offer us an opportunity to understand how cancer cells turn on its own death program in response to spindle poisons.

Antimitotic agents cause cells to arrest in the metaphase for some period of time prior to an aberrant exit from mitosis into a state called ‘mitotic catastrophe’. This activates a death pathway leading to cancer cell death, a feature contributing to the clinical response and prognoses of those drugs. The Bcl2 family of proteins, in particular, Mcl1 and Bcl-xL, have been implicated in the regulation of apoptosis from anti-mitotics in a number of different cancer types and models.^11–16^ However, how mitotic catastrophe turns on cell death machinery is still largely unknown.^17–19^ Here we provide evidence that antimitotic therapies activate a death receptor 3 (DR3)-mediated signaling pathway to kill cancer cells.

## Results

### Antimitotics induce caspase-8-dependent apoptosis

**Fig. 1:**
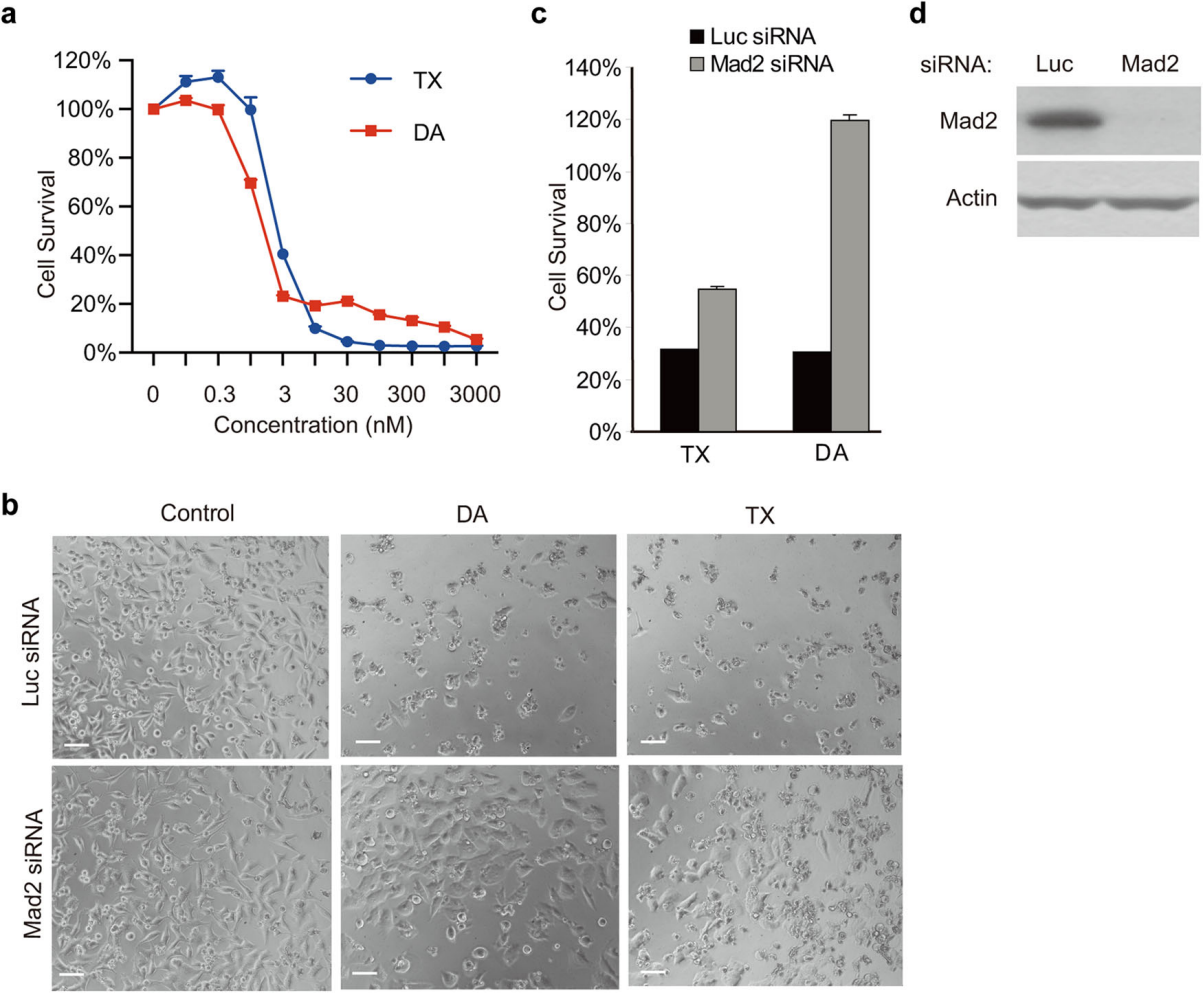
Depletion of spindle checkpoint blocks cell death induced by diazonamide or taxol. **a** Dose-dependent killing of HeLa cells by diazonamide (DA) or taxol (TX). Cells were treated with indicated concentrations of diazonamide or taxol. Cell viability was determined by measuring ATP levels using the CellTiter-Glo assay kit. In this and all the subsequent studies, cell survival was presented as the percentage of viable or dead cells compared with untreated control, and is represented as the mean ± s.d. of triplicates. **b**–**d** Effect of knockdown of spindle checkpoint protein Mad2 on diazonamide- or taxol- induced cell death. **b** HeLa cells were transfected with Luciferase siRNA or Mad2 siRNA for 24 h prior to incubation with 100 nM diazonamide or taxol for another 48 h. Phase-contrast images of cells are shown. Scale bars represent 100 µm. **c** Cell viability was determined after 48 h treatment of 100 nM diazonamide or taxol. **d** Mad2 knockdown efficiency was analyzed by western blotting with an anti-Mad2 antibody and normalized by Actin

We chose diazonamide to study anti-mitotics-induced cell death for three reasons. First, diazonamide exhibited a similar drug sensitivity pattern to taxol in killing a panel of cancer cell lines (Fig. 1a and Supplementary information, Figure S1). Second, after the spindle checkpoint was inactivated by knocking down Mad2, a component of spindle check point complex,^20^ cell death induced by diazonamide was completely blocked in human cervical carcinoma cells (HeLa). The cells continued to grow and became larger. However, knockdown of Mad2 only partially blocked taxol-induced cell death (Fig. 1b–d). This indicates that diazonamide-induced cell death solely depends on spindle checkpoint activation. Therefore, it can be used as a clean trigger to induce mitotic arrest. Third but importantly, diazonamide regresses tumors as efficiently as taxol, but displays safer profile than taxol in mouse xenograft models.^9,10^

**Fig. 2:**
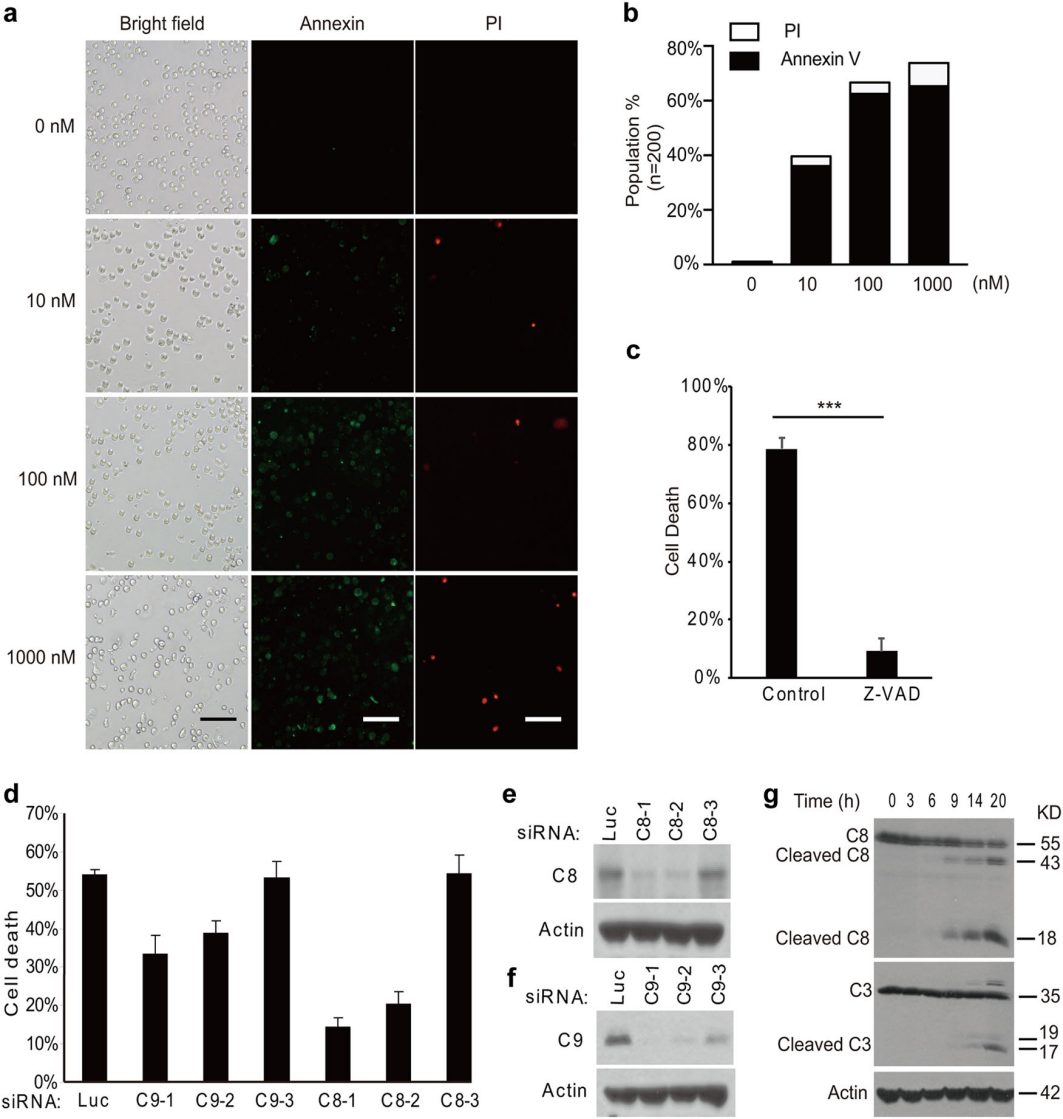
Antimitotics induce caspase-8-dependent apoptosis. **a** Fluorescent images of Annexin V/PI staining of HeLa cells treated with DMSO or indicated concentrations of diazonamide for 16 h. Scale bar: 100 μm. **b** The quantification of the cells positive for Annexin V or PI staining (*n* = 200). **c** HeLa cells were pretreated with 100 μM z-VAD for 2 h followed by 48 h treatment of 100 nM diazonamide (*p < 0.001). **d**–**f** HeLa cells were transfected with multiple siRNAs targeting caspase-8 or caspase-9. After 48 h, the cells were treated with 100 nM diazonamide for another 48 h. **d** Cell viability was determined by measuring ATP levels. **e**, **f** Whole-cell extracts were prepared followed by western blotting analysis for caspase-8 or caspase-9. **g** HeLa cells were treated with 100 nM diazonamide for the indicated time. Caspase activation, the hallmark of apoptosis, was determined by western blotting with anti-caspase-8 and anti-caspase-3 antibodies

Mitotic catastrophe may result in apoptosis, necrosis or senescence.^21^ In order to determine the type of cell death that is induced by anti-mitotics, HeLa cells were treated with different doses of diazonamide for 16 h and stained with Annexin V and Propidium Idodide (PI) to discriminate apoptosis from necrosis. The majority of cells were Annexin V staining-positive, while few cells were PI stained even at a compound dose as high as 1 μM (Fig. 2a, b). Furthermore, the cell death was largely blocked by a pan-caspase inhibitor z-VAD (Fig. 2c). These results indicate that anti-mitotics primarily induce apoptotic cell death.

Apoptosis in mammalian cells is activated via two major signaling pathways, extrinsic pathway and mitochondrial pathway, distinguishable by their initiator caspases—caspase-8 and caspase-9, respectively.^22,23^ Knocking down caspase-8 with multiple reported oligos^24,25^ caused attenuation of diazonamide-induced apoptosis. In contrast, knockdown of caspase-9, the initiator caspase for the mitochondrial apoptotic pathway, only slightly decreased cell death under the same condition. In both cases, the knockdown efficiency of the individual oligos correlated well with the protective effect (Fig. 2d–f). We then tested whether the extrinsic pathway was activated. As shown in Fig. 2g, diazonamide treatment led to caspase-8 cleavage and activation in 9 h, and caspase-3, the executioner protease, was evidently activated in 14 h.

### Antimitotics-induced apoptosis requires DR3

In efforts to elucidate the anti-mitotics-induced cell death, a high-throughput whole-genome siRNA library screen was performed to screen for genes whose knockdown would render cancer cells resistant to drug treatment in HeLa cells (Supplementary information, Data S1). HeLa cells showed high sensitivity to anti-mitotics (Fig. 1a), and high-efficiency of gene knockdown mediated by siRNA. Since mitotic arrest activated by spindle checkpoint preludes cell death induced by spindle poisons, we used a mitotic checkpoint protein Mad2 as the positive control in the screen. As shown in Fig. 1b–d, knockdown of Mad2 prevented HeLa cells from undergoing apoptosis induced by taxol or diazonamide. Because diazonamide treatment gave a distinct assay window between Mad2 RNAi and control RNAi (Fig. 1c), we chose diazonamide as the trigger of mitotic arrest for the screen.

**Fig. 3:**
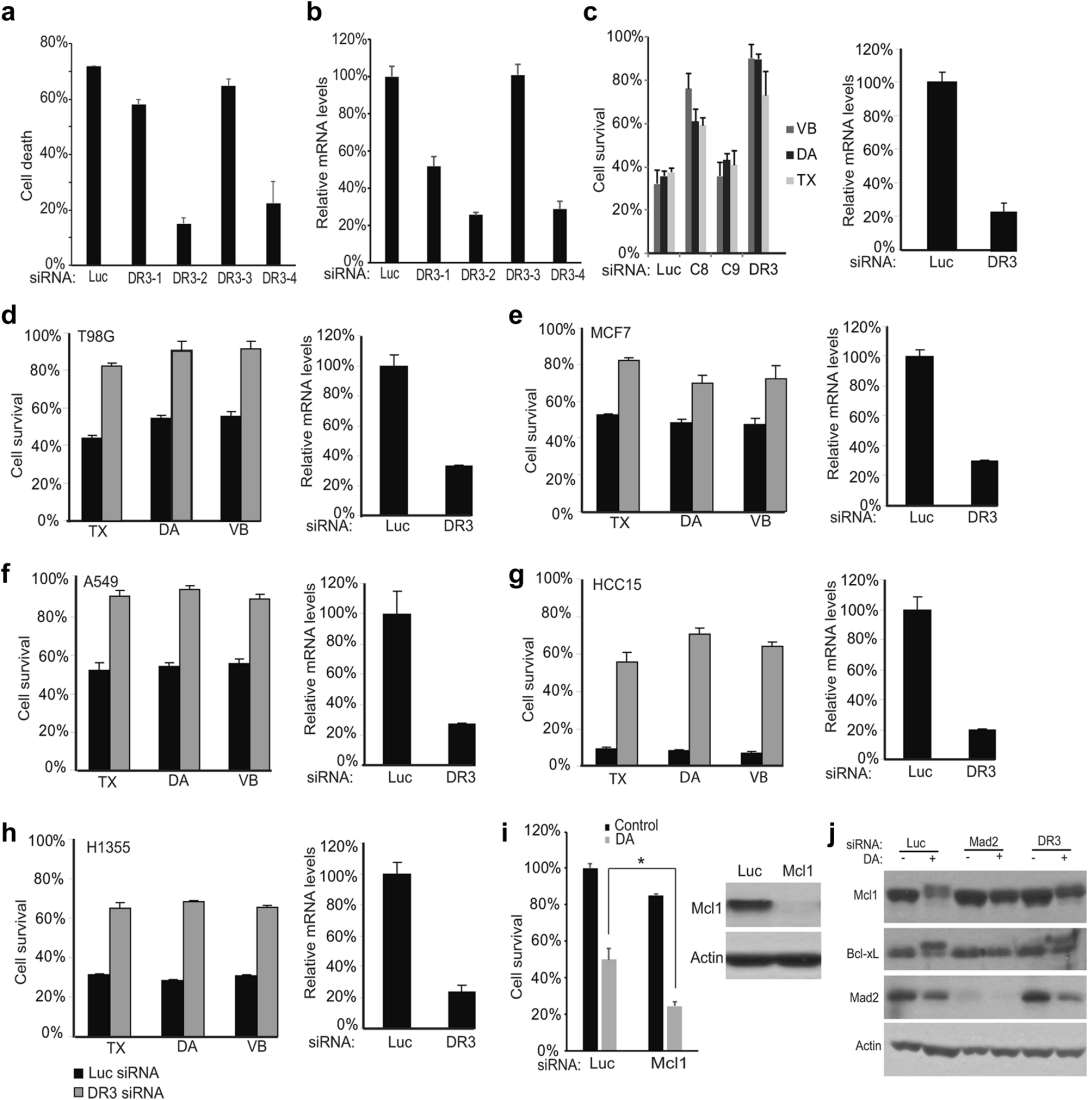
DR3 is necessary for apoptotic cell death. **a** HeLa cells were transfected with four DR3 siRNAs for 48 h. The cells were then treated with 100 nM diazonamide for another 48 h. The cell viability was determined by measuring ATP levels. **b** DR3 mRNA levels in knockdown cells were analyzed using quantitative RT-PCR. **c** HeLa cells were transfected with siRNAs targeting caspase-8, caspase-9 or DR3. After 48 h, the cells were treated with 100 nM diazonamide (DA), 100 nM taxol (TX) or 30 nM vinblastine (VB) for another 48 h. **d**–**h** DR3 is critical in initiating apoptosis induced by taxol, diazonamide or vinblastine in a variety of cancer cell lines. The cells were transfected with Luciferase or DR3 siRNA oligos followed by treatment with 100 nM diazonamide or taxol, or 30 nM vinblastine for 48 h. Cell viability (left panel) and DR3 mRNA levels (right panel) were determined. The following cancer cells were tested: (**d**) T98G (brain glioblastoma), (**e**) MCF7 (breast cancer), (**f**) A549 (lung cancer), (**g**) HCC15 (lung cancer), and (**h**) H1355 (lung cancer). **i** Knockdown of Mcl1 sensitizes HeLa cells to diazonamide. Mcl1 was knocked down in HeLa cells. Cell viability was determined by measuring cellular ATP levels (left panel) (*p < 0.05). The Mcl1 levels were measured by western blotting (right panel). **j** The effect of DR3 on the mitotic regulation of Mcl1 and Bcl-xL. Mad2 and DR3 in HeLa cells were knocked down followed by treatment with DMSO or diazonamide for 12 h. The cell lysates were analyzed by western blotting using antibodies as indicated

The screen hits mainly fell in six categories: spindle checkpoints, cell cycle regulators, apoptotic proteins, inflammatory signaling molecules, cytoskeleton proteins, and growth factors. An unanticipated hit from the siRNA library was death receptor 3, DR3. To confirm the role of DR3, we knocked down DR3 with four different siRNA oligos from the siRNA pool used in the screen. The four siRNA oligos knocked down DR3 to different degrees, and the knockdown efficiency correlated with the degree of apoptosis inhibition (Fig. 3a, b). To rule out the possibility that the cell survival effect of DR3 knockdown was due to any off-target effects of the siRNA oligos used, we engineered a HeLa cell line in which an shRNA targeting DR3 could be inducibly expressed by adding tetracycline. As shown in Supplementary information, Figure S2A-D, addition of tetracycline to the cell culture medium significantly increased cell survival in the presence of diazonamide. Such increase was countered by ectopic expression of an shRNA resistant DR3 transgene. Importantly, knockdown of DR3 not only allowed cell survival in diazonamide-treated cells, but also in cells treated with anti-tubulin drugs including taxol and vinblastine as well (Supplementary information, Figure S2E, F). We further measured the intracellular accumulation of these drugs in the tetracycline-inducible DR3shRNA cells by using the mass spectrometry methods. There was increase in vinblastine uptake or reduction in taxol and diazonamide uptake upon tetracycline addition (Supplementary information, Figure S2G), however, the knockdown or rescue effects on cells in response to these three compounds were similar. In addition, neither knockdown of DR3 by inducible shRNA nor expression of DR3 transgene had any effect on cell growth rate (Supplementary information, Figure S2H and I). Therefore, the potential effects of DR3 gene manipulation on drug uptake/retention or cell growth rate are not determining factors for the cell sensitivity to these drugs.

Death receptors such as TNFR1, TRAIL-R1/DR4, TRAIL-R2/DR5, FAS/CD95, are known to trigger apoptosis by activating intracellular caspase-8.^26,27^ We compared the magnitude of the rescue effects seen using siRNAs targeting caspase-8, caspase-9, and DR3 in HeLa cells (Fig. 3c). Caspase-9 knockdown had little if any rescue effect. DR3 knockdown showed better rescue effect than caspase-8 knockdown, suggesting that DR3 and caspase-8 act through the same pathway. Moreover, among six death-domain-containing death receptors and three decoy receptors, only knockdown of DR3, but not Fas, TRAIL receptors DR4 and DR5, TNFR1, DR6, DCR1, DCR2, or DCR3, increased cell survival in the presence of diazonamide (Supplementary information, Figure S3A-E). On the other hand, knockdown of DR3 had no effect on cell death induced by doxorubicin, a DNA-damaging agent (Supplementary information, Figure S3F).

The important role of DR3 in apoptosis was further confirmed in a panel of six cancer cell lines that are sensitive to anti-mitotics, including cervical cancer HeLa, glioblastoma T98G, breast cancer MCF7, and non-small cell lung cancer A549, H1355, and HCC15 cell lines. Knockdown of DR3 conferred resistance to all these cells upon the treatment of diazonmaide, taxol or vinblastine (Fig. 3c–h).

During mitotic catastrophe, CDK1­mediated phosphorylation inhibits Bcl­xL and destabilizes Mcl1 to facilitate cell death,^13,28,29^ indicating the contributing role of the mitochondrial pathway. As expected, knockdown of Mcl1 slightly decreased cell viability, and sensitized the cells to diazonamide (Fig. 3i). To delineate DR3 and Bcl2 family proteins in cell death triggered by anti-mitotics, we examined the regulation of Mcl1 and Bcl-xL in response to diazonamide in Mad2 or DR3 knockdown cells. As shown in Fig. 3j, the Mcl1 degradation/phosphorylation or Bcl-xL phosphorylation is largely blocked when Mad2 is eliminated, and partially blocked when DR3 is depleted, suggesting that Mad2 and DR3 may act upstream of Mcl1 and Bcl-xL.

### Ectopic expression of DR3 converts cellular response from mitotic arrest to apoptosis

**Fig. 4:**
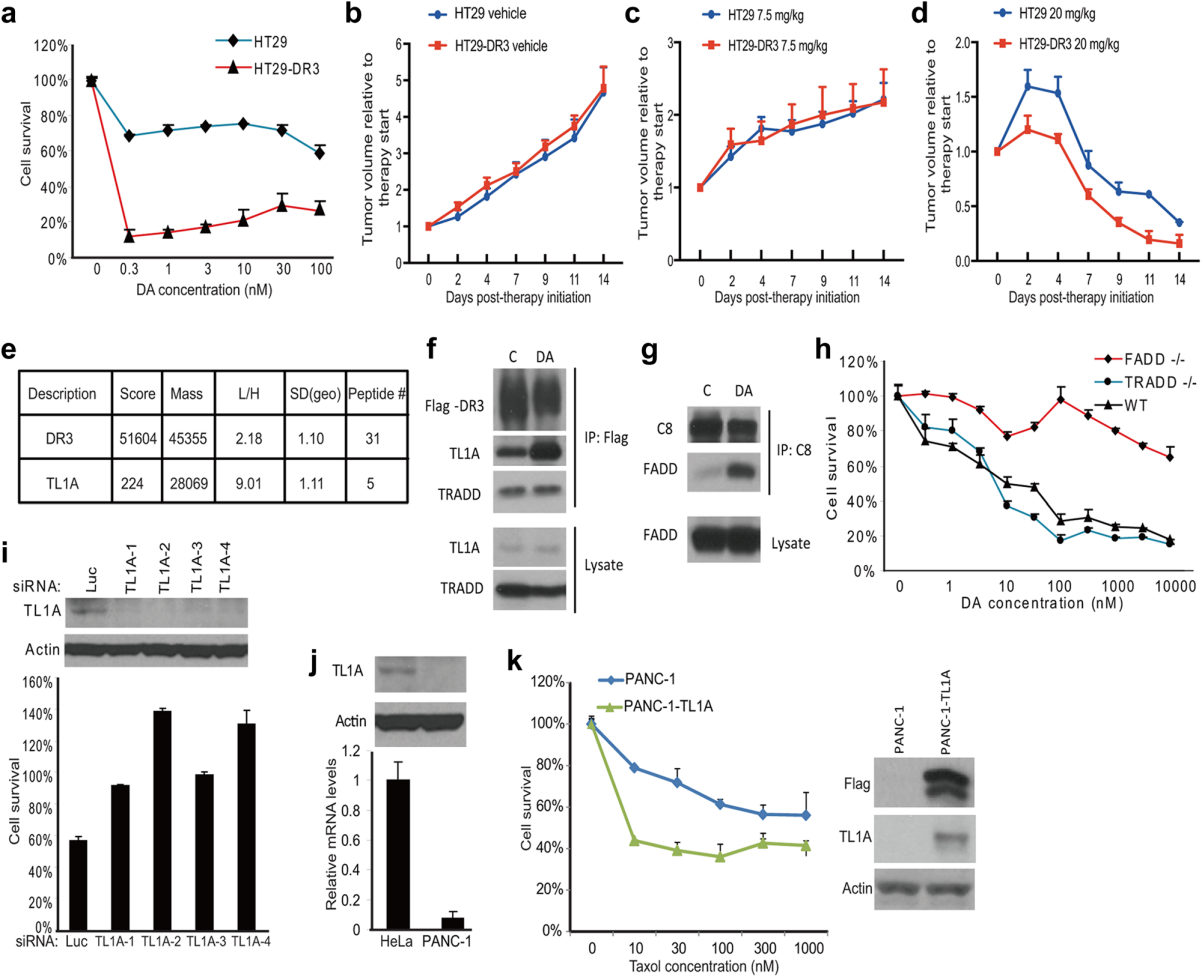
The ligand of DR3, TL1A, is required for DR3-mediated apoptosis. **a** The cell line stably expressing Flag-tagged DR3 was established in HT29 cells. Dose response curves of HT29 cells and HT29-DR3 cells to diazonamide were plotted. Cell viability was measured after 48 h of treatment. **b**–**d** HT29 or HT29-DR3 tumor-bearing mice were treated with vehicle (**b**), 7.5 mg/kg taxol (**c**), or 20 mg/kg taxol (**d**) for the indicated time. Tumor growth was monitored. Error bars, SEM. **e** Identification of TL1A as the only TNF family death ligand bound to DR3. A SILAC experiment was carried out to quantify the DR3 binding proteins as described in the Materials and Methods. HT29-DR3 cells growing in the light medium were treated with 100 nM diazonamide for 12 h. Untreated cells were grown in the heavy medium. Equally combined cell lysates were subjected to immunoprecipitation with an anti-Flag antibody. Proteins in the whole elution with 3× Flag peptide were quantified and the Light/Heavy ratios were calculated. SD (geo) stands for geometric s.d. The closer the SD (geo) is to 1, the better the protein quantification is. The median from peptide ratios was used as the protein ratio. (**f**) HT29-DR3 cells were treated with 100 nM diazonamide for 12 h. The DR3/TL1A complex was analyzed by Flag immunoprecipitation followed by western blotting with antibodies against Flag, TL1A, and TRADD. **g** HT29-DR3 cells were treated as in **f**. The DISC complex was analyzed by caspase-8 immunoprecipitation followed by western blotting with antibodies against caspase-8 and FADD. **h** The dose response curves of wild-type MEFs, FADD-/- MEFs, and TRADD-/- MEFs to diazonamide. **i** Knockdown of TL1A blocked anti-mitotics-induced apoptosis. HeLa cells were transfected with four TL1A siRNAs for three rounds before being treated with 100 nM diazonamide for 48 h. Cell viability was determined (lower panel). Protein levels of TL1A in the knockdown cells were determined by western blotting with an anti-TL1A antibody (upper panel). **j**, **k** Exogenous expression of TL1A sensitizes PANC-1 cells to taxol. **j** The relative TL1A mRNA levels in HeLa and PANC-1 cells were determined by qRT-PCR (lower panel). TL1A protein levels in HeLa or PANC-1 cells were determined by western blotting with anti-TL1A and anti-Actin antibodies (upper panel). **k** Flag-tagged TL1A was stably expressed in PANC-1 cells. Dose response curves to taxol were plotted for parental PANC-1 cells and PANC-1-TL1A cells. Cell viability was measured after 48 h treatment (left panel). Cell lysates from PANC-1 and PANC-1-TL1A cells were subjected to western blotting with anti-Flag, anti-TL1A and anti-Actin antibodies (right panel)

DR3 (Apo3, TRAMP, LARD, WSL-1, TNFRSF25) is predominantly expressed by T lymphocytes and to a much less extent by other types of cells.^30,31^ Human colorectal cancer cell line HT29 is one of the cancer cell lines that have undetectable DR3 expression.^32^ HT29 cells were reported to be resistant to taxol and a small-molecule inhibitor of Eg5 (also a spindle poison),^33^ and our data was consistent with this observation when HT29 cells were treated with diazonamide or taxol for 48 h (Supplementary information, Figure S4A). In contrast, derivatives of the parental HT29 cells that expressed a DR3 transgene readily underwent apoptosis in the presence of as low as 0.3 nM diazonamide while DR3 overexpression had little effect on cell growth rate (Fig. 4a and Supplementary information, Figure S4B). The same cells also showed dramatically increased sensitivity to taxol and vinblastine (Supplementary information, Figure S4C and D). The contribution of DR3 to the increased sensitivity of HT29 cells to antimitotic agents was further confirmed by the loss of sensitivity when the expression of DR3 transgene was knocked down by multiple siRNA oligos. Moreover, the level of sensitivity correlated well with the degree of DR3 knockdown (Supplementary information, Figure S4E). Both HT29 and HT29-DR3 cells treated with taxol showed sustained cell cycle arrest at the G2/M phase as judged by their cell cycle profiles (Supplementary information, Figure S4F). To determine whether HT29-DR3 tumors respond better than HT29 tumors to taxol in vivo, HT29 and HT29-DR3 xenograft-bearing nude mice were treated with vehicle, taxol at 7.5 mg/kg or 20 mg/kg three times per week for 2 weeks. The xenograft tumors progressed similarly in the vehicle control, and the tumor growth was inhibited similarly in both models at low dose of taxol. Nevertheless, the HT29-DR3 xenografts in mice displayed faster tumor regression than the HT29 xenografts when treated with high dose of taxol (Fig. 4b–d). These results indicate that the apoptotic response can be reconstituted by ectopic expression of DR3 in HT29 cells.

To test whether DR3 overexpression in HT29 cells might alter general apoptosis sensitivity, HT29-DR3 cells were treated with six other toxins. HT29-DR3 cells were found to show slightly increased sensitivity to actin depolymerizing agent, cytochalasin D. Little or no sensitizing effect was observed with other toxins, such as clinical therapeutic proteasome inhibitor, DNA-damaging agent, mTOR inhibitor, and ER stressor (Supplementary information, Figure S5). As such, DR3 overexpression appears to selectively render cells sensitive to drugs that perturb cytoskeleton, especially microtubules.

### Identification of TL1A as the responsible ligand that activates DR3

The extrinsic pathway is triggered from the exterior of the cell by members of the tumor necrosis factor (TNF) superfamily of cytokines, which bind to and activate their corresponding death receptors. The ligand-receptor complex engages the adapter protein Fas-associated death domain (FADD). FADD then recruits the initiator caspase-8 to form a death-inducing signaling complex (DISC). DR3 is one of the least characterized cell death receptors and multiple potential ligands have been proposed for DR3 including Tweak and TNF like 1a (TL1A, also known as TNFSF15 and VEGI).^34–36^ Follow-up studies indicate that Tweak can signal without DR3,^37^ and that Fn14 (TNFRSF12A) was identified as Tweak receptor.^38^ HT29-DR3 cell line allowed us to probe DR3 protein with Flag tag in the biochemical study of the pathway. To search for the DR3 ligand that specifically activates DR3 in response to the antimitotic agents, we performed a stable isotope labeling (SILAC) experiment to identify interacting partner of DR3 when the cells were treated with diazonamide for 12 h. As shown in Fig. 4e, although DR3 levels were only modestly increased by two folds, TL1A, one member of the TNF family of cytokines, showed a ninefold increase in association with DR3 in diazonamide versus vehicle-treated cells. TL1A was the only member of the family that was co-immunoprecipitated with antibodies to the cell surface receptor DR3. The specific increase of the interaction between TL1A and DR3 after treatment with diazonamide, taxol, or vinblastine was confirmed by western blotting analysis using an antibody against TL1A (Fig. 4f and Supplementary information, Figure S6A). By contrast, these drugs did not alter the recruitment of TNFR1-associated death domain protein (TRADD) to DR3. At this time point of treatment, FADD was undetectable on the DR3 immunocomplex, but rather recruited downstream effector caspase-8, which triggers cell death (Fig. 4g and Supplementary information, Figure S6B). In line with this observation, FADD-/- MEFs displayed much less sensitivity to diazonamide than wild-type MEFs or TRADD-/- MEFs (Fig. 4h), supporting the role of FADD-mediated formation of death-inducing signaling complex (DISC) in anti-mitotics-induced apoptosis.

The importance of TL1A in anti-mitotics-induced apoptosis was further verified by RNAi-mediated knockdown of TL1A expression. As shown in Fig. 4i, knockdown of TL1A prevented cell death induced by diazonamide in HeLa cells. This rescue effect was recapitulated in the same panel of sensitive cell lines used for DR3 knockdown (Supplementary information, Figure S7). Like DR3, TL1A expression also varies from cell to cell. For example, HeLa cells express about 10-fold more TL1A than human pancreatic cancer cells PANC-1 (Fig. 4j). PANC-1 cells turned out to be resistant to taxol treatment (Supplementary information, Figure S8A). On the other hand, PANC-1 cells expressing a TL1A transgene showed corresponding sensitivity to taxol-induced apoptosis, but not to other stimuli tested (Fig. 4k and Supplementary information, Figure  S8B-C).

### Autocrine TL1A activates DR3

Since DR3-TL1A signaling appears to be required in anti-mitotics-induced apoptosis, by analogy to the closest relative, TNFR1-TNFα signaling,^24^ we reasoned that these cells might be secreting TL1A into the culture medium. Quantitative ELISA analysis of the condition media from HeLa cells revealed a significant increase of TL1A secretion upon treatment of taxol, vinblastine, or diazonamide (Supplementary information, Figure S8D-E), whereas TL1A protein levels were unaffected (Fig. 4f and Supplementary information,  Figure  S6A). In contrast, several other ligands for TNF receptor superfamily such as TNFα, FasL, or TRAIL were undetectable in the condition medium from the cells treated with diazonamide under the same condition. In addition, cell imaging of Flag-tagged DR3 did not show any enhanced surface representation or decreased endocytosis of DR3 (Supplementary information, Figure S9). These observations suggested that cell death in response to anti-mitotics might be stimulated by toxin-mediated increases in TL1A secretion.

**Fig. 5:**
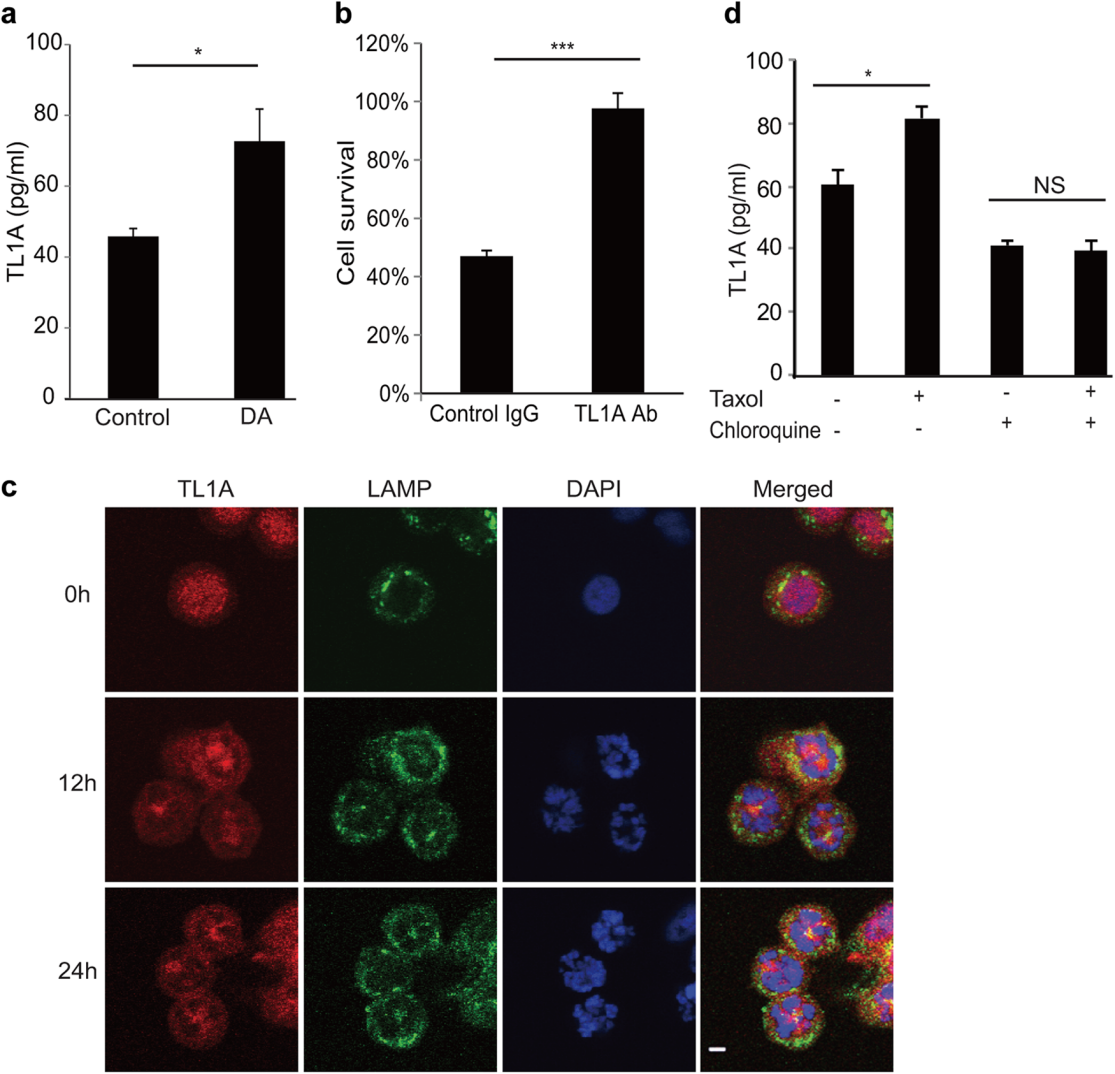
Autocrine TL1A activates DR3. **a** HT29 cells were treated with or without 100 nM biotinylated diazonamide for 16 h before the conditioned cell culture media were removed for soluble TL1A analysis. A TL1A ELISA kit was used to determine the concentration of TL1A released into the media. **b** HT29 cells were treated as in **a**. Then biotinylated diazonamide was eliminated from condition medium by streptavidin agarose. The concentrated medium was applied to the HT29-DR3 cells pretreated with control IgG or TL1A neutralizing antibody. Values are presented as means ± s.d. **c**, **d** DR3 secretion is lysosome-dependent. **c** HT29-DR3 cells were grown in the chamber slides and treated with 100 nM taxol for the indicated time. Cells were fixed and stained with anti-TL1A and anti-LAMP2 antibodies. The slides were mounted in a medium containing DAPI to stain DNA (Scale bar: 5 μm). **d** HT29-DR3 cells were treated with 100 nM taxol in the presence or absence of 100 μM chloroquine for 16 h. TL1A ELISA kit was used to measure the concentrations of TL1A in the medium. *p < 0.05, ***p < 0.001

The above results raise the possibility that antimitotic agents shift cellular response from mitotic arrest to apoptosis by inducing autocrine TL1A that activates DR3. To directly verify this, we took cell culture medium from HT29 cells treated with biotinylated diazonamide, which was removed by streptavidin beads before applying the condition medium to naive HT29-DR3 cells. As shown in Fig. 5a, diazonamide treatment resulted in an elevated TL1A secretion in HT29 cells, but the cells did not respond because of lack of the receptor. However, HT29-DR3 cells readily underwent apoptosis when exposed to the condition medium from diazonamide-treated HT29 cells. The role of TL1A in the culture medium in inducing apoptosis was confirmed by adding the TL1A neutralizing antibody to the culture medium as such TL1A neutralizing  antibody blocked apoptosis (Fig. 5b). However, co-treatment of the TL1A neutralizing  antibody did not suppress cell death induced by anti-mitotics in HeLa cells.

Having observed that antimitotic agents induced autocrine TL1A, we wonder whether this secretion might be through lysosomal exocytosis. To this end, cultured HT29-DR3 cells were exposed for 12 and 24 h to taxol and stained with antibodies recognizing TL1A and lysosome marker LAMP2. As shown in Fig. 5c, most untreated cells were in the interphase and TL1A localized in nucleus and cytosol. Taxol treatment led to cells arrested in mitosis with disorganized and condensed chromosome. TL1A migrated to colocalize with lysosome. Chloroquine raises the lysosomal pH to prevent endosomal acidification and lysosomal function. Treatment of HT29-DR3 cells with chloroquine blocked TL1A secretion (Fig. 5d).

**Fig. 6:**
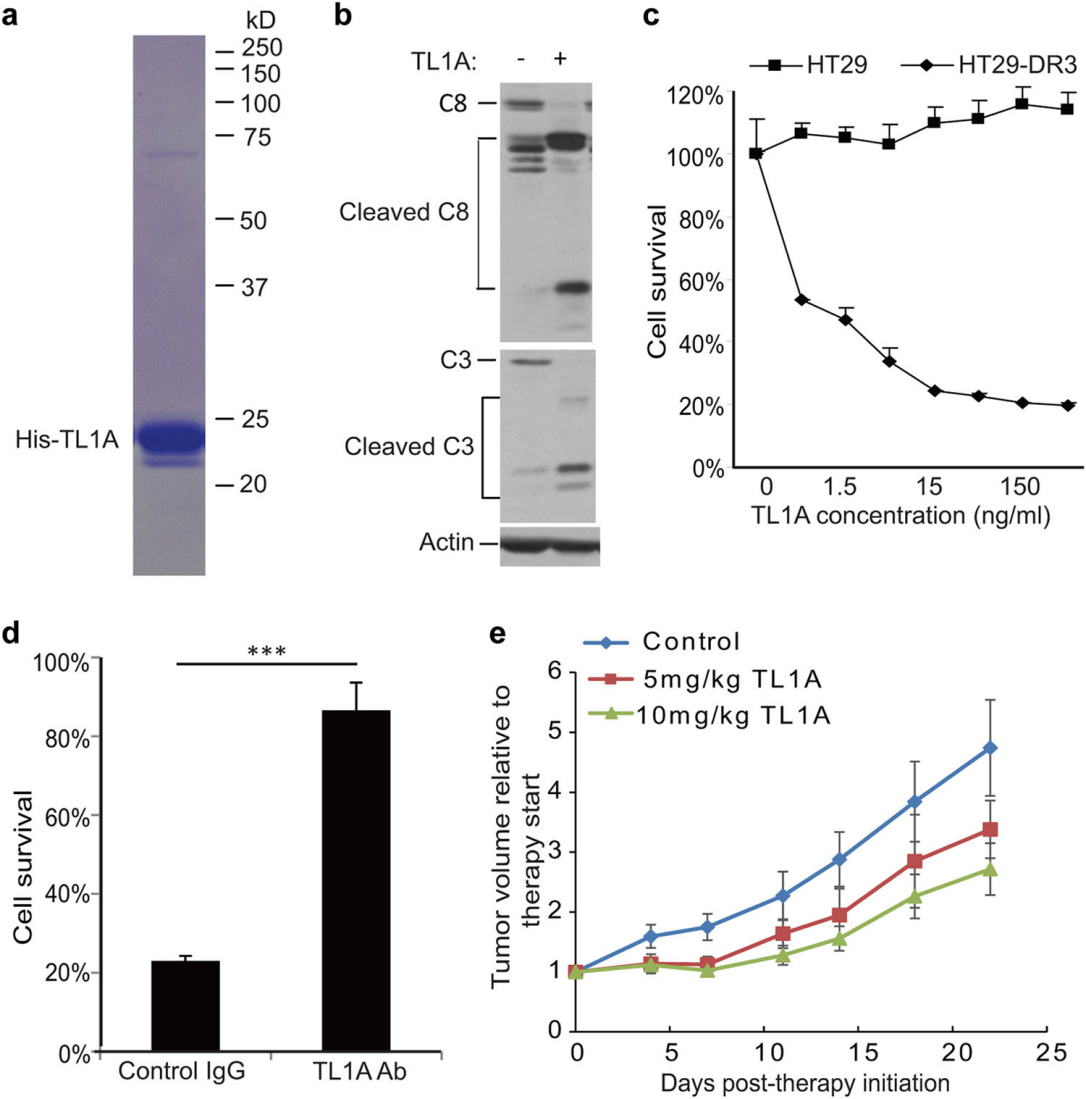
Pro-apoptotic activity of recombinant TL1A against HT29-DR3 cells. **a** Purified recombinant His-tagged TL1A on the coomassie blue stained SDS-PAGE gel. **b** HT29-DR3 cells were treated with 100 ng/ml His-TL1A for 24 h. Cell lysates were subjected to western blotting analysis of caspase-8 and caspase-3 activation. **c** HT29 cells and HT29-DR3 cells were treated with the indicated concentrations of His-TL1A. After 72 h, cell viability was measured. **d** HT29-DR3 cells were co-treated with 100 ng/ml His-TL1A and 2 μg/ml TL1A neutralizing antibody for 72 h. Values are presented as means ± s.d. (***p < 0.001). **e** Relative tumor growth curves of xenografts derived from HT29-DR3 cells. Athymic nude mice were injected subcutaneously with HT29-DR3 cells and then randomly separated into three treatment groups: control (*n* = 6), TL1A at 5 mg/kg (*n* = 4), or 10 mg/kg (*n* = 5). The mice were given six intravenous injections of TL1A or saline every other day. Tumors were measured twice per week until the end of the experiment

The observed apoptosis was recapitulated with purified recombinant TL1A (Fig. 6a, b). HT29-DR3 cells increasingly underwent apoptosis with the increased amount of recombinant TL1A added to the cell culture medium, whereas the parental HT29 cells did not respond to TL1A at all (Fig. 6c). The action of recombinant TL1A could be countered by TL1A neutralizing antibody (Fig. 6d). Recombinant TL1A triggered apoptosis in DR3-expressing tumor cell lines in combination with a protein synthesis inhibitor cycloheximide.^34^ Moreover, the injection of recombinant TL1A led to a much slower progression of mouse xenograft tumors derived from HT29-DR3 cells in a dose-dependent manner (Fig. 6e). The concentration of endotoxin was 0.35 EU/mg protein in recombinant TL1A. For a 20 g mouse, the maximal dose (10 mg/kg) of TL1A contained 0.07EU endotoxin, which is within the safety range (<0.1 EU/h). However, recombinant TL1A did not enhance cell death in HeLa cells treated with anti-mitotics (Supplementary information, Figure S8F-H), suggesting that the secretion of TL1A induced by anti-mitotics is sufficient to trigger apoptosis.

### Expression of DR3 and TL1A correlates with the apoptotic response of cancer cells

**Fig. 7:**
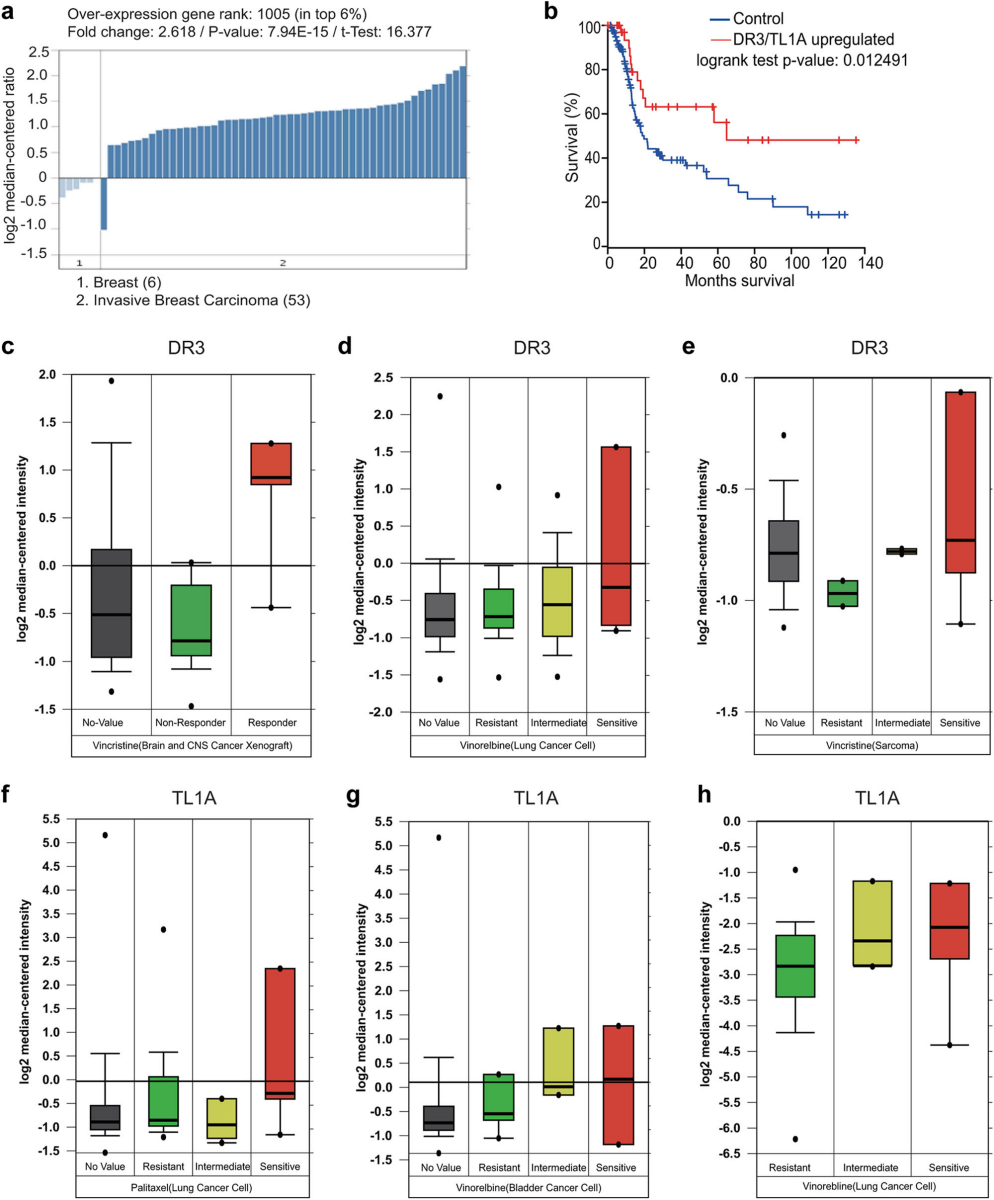
Expression of DR3 and TL1A correlates with the apoptotic response of cancer cells. **a** TL1A is overexpressed in most breast cancers. The Oncomine analysis was performed in six normal breast tissues and 53 invasive breast carcinomas. **b** Breast cancer patients with higher expression of DR3 or TL1A show better prognosis. Kaplan–Meier survival curves were generated from The Cancer Genome Atlas using the invasive breast cancer datasets. **c**–**h** Box plot showing the significant association between DR3 (**c**–**e**) and TL1A (**f**–**h**) expression and the response to antimitotic drugs (Vincristine, Vinorelbine, Paclitaxel) in multiple cancer types (brain, lung, sarcoma, and bladder cancers). Data have three sources. First, the Pediatric Preclinical Testing program (PPTP) using human tumor xenograft models and derived cell lines; second, a large-scale screen using 639 human cancer cell lines that aims to identify drug-sensitivity biomarkers to a broad range of cancer drugs;^41^ third, expression signatures that associate with metastasis and resistance to chemotherapy in 27 initial tumor biopsies of Ewing’s sarcoma family of tumors (ESFT).^42^ Data were mined through the Oncomine website. The response to Vincristine in sarcoma cancer cells can be found at: https://www.oncomine.org/resource/ui/component/dataset.html?component=d:97761287. All other data can be found at: https://www.oncomine.org/resource/ui/component/dataset.html?component=d:156636665

Genome-scale technologies, analytical tools and unprecedented amount of valuable clinical information drive therapeutic, diagnostic and prognostic advances in cancer medicine.^39^ Because DR3 and TL1A expression appeared to be important in determining the apoptotic response of cancer cells in our laboratory studies, we sought to expand our finding by mining vast database offered by Oncomine. As shown in Supplementary information, Figure S10, DR3 is differentially expressed in various cancers. It is overexpressed in bladder cancer, cervical cancer, gastric cancer, leukemia, lymphoma, but its expression is decreased in melanoma, prostate and colon cancer. In addition, for the above-mentioned cancers with high DR3 expression, the corresponding normal tissue has relatively lower DR3 expression. TL1A is overexpressed in most breast cancers and some of the colon cancers. We chose to look at breast cancer for the association of DR3/TL1A expression with patient survival for the following reasons. First, TL1A is overexpressed in most breast cancers (Fig. 7a). Second, the anti-tubulin drugs are the standard-of-care for the patients with breast cancer. Third, the role of DR3/TL1A in apoptosis caused by anti-mitotics has been implicated in a breast cancer cell line MCF7 in this study. The Kaplan–Meier survival curves were generated from *The Cancer Genome Atlas* using the invasive breast cancer datasets. Patients with higher expression of DR3/TL1A showed better prognosis. These patients showed remarkable overall survival advantage versus the rest of the cohort with ~50% of those patients still alive at the end of the study (140 month), whereas less than 20% of the rest of the cohort still alive at that time point (Fig. 7b).

The concept of TL1A/DR3-mediated cancer cell death in response to anti-tubulin drugs is substantiated by mining clinical datasets from three resources. First, the Pediatric Preclinical Testing program (PPTP) using human tumor xenograft models and derived cell lines. The xenograft models and cell lines accurately recapitulated the expression profiles of their corresponding primary tumor samples.^40^ Second, a large-scale screen using 639 human cancer cell lines that aims to identify drug-sensitivity biomarkers to a broad range of cancer drugs.^41^ Third, expression signatures associated with metastasis and resistance to chemotherapy in 27 initial tumor biopsies of Ewing’s sarcoma family of tumors (ESFT).^42^ We found a significant association between DR3 and TL1A expression and response to vincristine, vinorelbine and paclitaxel in multiple cancer types (brain, lung, sarcoma, and bladder cancers; Fig. 7c–h). This observation suggests that combined expression of DR3 and TL1A might be used as a predictive marker of responsiveness when patients are subjected to chemotherapy with anti-tubulin drugs such as vincristine, vinorelbine and paclitaxel.

## Discussion

Mitosis has been clinically utilized as a successful anticancer target for more than a half century. Such drugs that arrest cells in mitosis are established treatment or important components of combination chemotherapy regimens for a variety of human cancers such as breast cancer, ovarian cancer, neuroblastoma, head and neck cancer, non-small-cell lung cancer, testicular cancer, Hodgkin’s disease, and acute lymphocytic leukemia.^1^ These drugs cause defects in the mitotic spindles that activate spindle checkpoint to arrest the cell cycle. Long-term mitotic arrest leads to apoptosis. The signaling pathways that activate cell death induced by antimitotic chemotherapeutics are of great interest. Earlier studies emphasized the contribution of the mitochondrial pathway because some pro-survival Bcl2 family proteins were reported to be important regulators of this therapeutic response. Prolonged mitotic arrest is known to lead to GSK-3-mediated phosphorylation and an E3 ubiquitin ligase FBW7-mediated degradation of Mcl1.^13,43^ However, it is under debate whether mitotic degradation of Mcl1 is under the control of an E3 ligase.^12^ Bcl-xL and proapoptotic BH3 protein Bak, are implicated as the other key regulators of apoptosis in mitotic arrest.^15,44^ Mitotic arrest by anti-tubulin drugs is also reported to produce a temporally controlled DNA damage response (DDR) characterized by the caspase-dependent formation of γH2AX foci in non-apoptotic cells, and is also controlled by Bcl2 family proteins.^16^ A few years ago, we performed a genome wide siRNA screen to search for genes responsible for anti-mitotics-induced cell death in an unbiased manner. From there, we identify death receptor DR3 as the essential factor that mediates apoptosis in response to two mechanistically distinct anti-tubulin chemotherapeutics (taxol and vinblastine), and a new spindle poison (diazonamide). The suppression of mitotic phosphorylation/degradation of Mcl1 and phosphorylation of Bcl-xL in DR3 knockdown cells suggests DR3 is epistatic in the apoptotic pathway, and a crosstalk occurs when caspase-8 activation leads to cleavage of Bid, a Bcl-2-interacting protein that activates the mitochondrial pathway.^45^ We now hypothesize that anti-mitotics stimulate autocrine secretion of a TNF family member of death ligand TL1A, which is subsequently used as the extrinsic death signal by cancer cells. Engagement of secreted TL1A with its receptor DR3 activates caspase-8-dependent apoptotic pathway. In addition to offering progress towards an understanding of anti-mitotics-induced cell death, this work points to an important direction to further investigate the underlying mechanism by finding the missing link between mitotic catastrophe and TL1A secretion.

DR3 is one of the six members of death-domain-containing TNFR family proteins that also include TNFR1, Fas, DR4, DR5 and DR6. Like the closest relative TNFR1, DR3 binds the adapter molecule TRADD. TRADD recruitment is thought to endow DR3 the dual power of activating NF-kB or triggering caspase activation and apoptosis.^30,31^ TNFR1 is expressed ubiquitously, whereas DR3 transcripts are present mainly in spleen, thymus, and peripheral blood and are induced by activation in T cells.^27^ Our data suggest that pre-existing DR3-TRADD complex in DR3 overexpressing cells is not capable to transduce the death signal to caspase-8, whereas cognate ligand TL1A secretion through lysosome and binding to DR3 upon anti-mitotics treatment promote DR3 clustering and subsequent FADD recruitment. FADD then recruits caspase-8 to form the death-inducing signaling complex (DISC) to execute apoptosis.

Although antimitotic therapy appears to be broad spectrum since it targets an essential biological process in all cancer cells, clinical responses to this treatment are often limited to an undefined subset of patients. Alteration in cancer genome may explain some of the variation in the responsiveness. However, the genomic associations for the anti-tubulin chemotherapeutics are generally less significant than for targeted drugs.^41^ A generalized mechanism of sensitivity to these drugs has not been well documented. Our loss-of-function and gain-of-function studies of DR3/TL1A suggest that the susceptibility of cancer cells to tubulin-targeting agents is determined by the combination expression of TL1A and its receptor DR3, offering some explanation for the tissue specificity of these drugs. Our in vitro and in vivo studies indicate that overexpression of DR3/TL1A can enhance killing of cancer cells and regression of xenograft tumors. This offers the possibility of transforming non-responsive cancer cells to sensitive ones by manipulation of the DR3/TL1A expression, thus expanding the applications of this established cancer treatment regimen.

DR3 is a death receptor primarily expressed on T cells. In contrast to relatively unexplored proapoptotic role of DR3, the physiological function of DR3-TL1A interaction in immunity is well studied. TL1A functions as a T-cell costimulator of T-cell proliferation and proinflammatory cytokine production using DR3 as its cognate receptor.^34,35^ Antimitotic drugs are known to have side effects such as neutropenia and immune suppression. This work points to several important, unanswered questions. First, with a relatively low proliferation rate, may T cells be as susceptible as cancer cells to anti-mitotics? Second, does activation of DR3/TL1A pathway lead to enhancement or suppression of cancer immunity in response to anti-mitotics? That is, does anti-mitotics induce DR3-mediated apoptosis of T cells, or proliferation and activation of T cells? Third, how can the selectivity of antimitotic drugs be attributed to the differential expression of DR3 in cancer versus normal cells?

There is intense controversy on how antimitotic drugs successfully exert antitumor activity in patients. First, this class of drugs induce mitotic arrest, whereas cell death occurs in interphase.^46^ Consistent with this report, we observed that after prolonged mitotic arrest, many cells underwent aberrant mitotic exit into interphase without cytokinesis and assembled abnormal interphase multi-nuclei. Second and foremost, unlike in tissue culture where cells divide every 20–50 h, in patients, tumor’s doubling times are in the order of tens to hundreds of days.^47^ Our finding that anti-tubulin drugs induce activation of DR3 by autocrine TL1A raises an interesting possibility that these drugs not only directly stop tumor cells from dividing, but also cause them to undergo apoptosis that might ultimately contribute to the efficacy of those therapies.

## Materials and methods

### General reagents

Z-VAD was purchased from Calbiochem. The antibodies used were as follows: anti-caspase-3 (Cell signaling, 9662), anti-caspase-8 (Cell signaling, 9746), anti-caspase-8 (Santa Cruz Biotech, SC-6136), anti-caspase-9 (Cell signaling, 9508), anti-TRADD (Cell signaling, 3684), anti-actin (Sigma, A2066), anti-Flag (Sigma, F-3165), anti-tubulin (Sigma, T7816), anti-PARP (Cell signaling, 9542), anti-Mcl1 (BD, 554103), anti-TL1A (Enzo, ALX-804-859-C100), and anti-TL1A (PEPROTECH, 500-P240). Recombinant proteins: FasL (R&D, 126-FL/CF), TNFα^24^ and TRAIL^25^. ELISA kits: TL1A (ENZO Life Science, APO-54N-027), TNF-alpha (RayBiotech, ELH-TNFα), FasL (RayBiotech, ELH-FASL), and TRAIL (RayBiotech, ELH-TRAIL).

### Plasmids and siRNA transfection

In general, plasmid transfections were performed using Lipofectamine 2000 reagent, while siRNA transfections were done using Lipofectamine RNAiMAX reagent following manufacture’s instruction (Invitrogen). For reverse siRNA transfections in 96-well plate, 5 pmol siRNA and 0.25 μL RNAiMAX reagent were used to transfect 5000 cells in each well.

### Generation of stable cell lines

Full-length *DR3* or *TL1A* cDNA with 3 × Flag at the C-terminus was cloned into retroviral vector pMXs-IRES-Blasicidin (Cell Biolabs). Human colon adenocarcinoma cell line HT29 was infected with the viral suspension. Cells were grown in DMEM containing 1 μg/ml blasticidine. After 2 to 3 weeks, clones were picked up and checked for exogenous DR3 expression using western blotting with an anti-Flag antibody.

### Preparation of recombinant protein

cDNA corresponding to the TL1A (amino acids 72–251) was cloned into pET28a to produce the fusion protein with 6× His tag at both N-terminus and C-terminus. The recombinant protein was expressed in *E. coli* BL21 (DE3) strain and purified using Ni-NTA agarose beads (Qiagen) and Hi-trap Q column.

### PI and Annexin V staining assay

Cells were cultured in six-well plates at a density of 1.2 × 10^5^ cells/well and allowed to adhere to culture plate overnight. Cells were treated with DMSO or diazonamide at 10, 100, or 1000 nM for 16 h. The cells were then trypsinized by EDTA-free trypsin and washed twice with cold PBS. Aliquots of the cells were re-suspended in 100 μL of binding buffer and stained with 1 μL of annexin V-FITC and 1 μL of PI working solution for 15 min at room temperature in the dark (DOjindo). The cell images were taken under an inversed fluorescence microscope (Olympus).

### ELISA analysis of TNF ligands

Cells were grown in six-well plates. Media was removed on the next day. Fresh media with or without 100 nM diazonamide, 100 nM taxol, or 30 nM vinblastine was added. After 16 h treatment, 100 μL aliquots were removed to measure TL1A concentration in the media. Three independent wells were tested. The secretion of TNF ligands was measured in a sandwich Enzyme-Linked Immuno Sorbent Assay (ELISA) using an ELISA kit following manufacturer’s manual.

### Xenograft studies

#### In vivo regression of HT29 and HT29-DR3 tumor models by taxol

A total of 7.5 × 10^6^ HT29 and HT29-DR3 cells in a volume of 0.2 mL were implanted in a subcutaneous site in the right flank of nu/nu mice. When tumors reached 280 mm^3^, the mice were randomized into a vehicle control group and two treatment groups (taxol at 7.5 mg/kg and 20 mg/kg), so that the average tumor volume is similar in all groups at the start. Mice in the control and treatment cohorts (*n* = 6–8) were subsequently treated with drugs by intravenous delivery three times per week. Taxol was first dissolved in ethanol at 5% of total volume, then 5% of final volume of cremophor and finally 90% of final volume of D5W (5% dextrose in water, pH 7.4) were added. All animals were cared for in accordance with the animal care and use policy of the Tsinghua University.

#### In vivo regression of HT29-DR3 tumor model by TL1A

Athymic nude mice were injected subcutaneously with HT29-DR3 cells as described above, and then randomly separated into three treatment groups: control (*n* = 6), TL1A at 5 mg/kg (*n* = 4), or 10 mg/kg (*n* = 5). The mice were given six intravenous injections of TL1A or saline every other day. Tumors were measured twice per week until the end of the experiment. All these mouse experiments were approved and performed in accordance with the Institutional Animal Care and Use Committee (IACUC) at the University of Texas Southwestern Medical Center.

### Statistics

*P-*values were obtained by *t*-test, paired two samples for mean.

## Electronic supplementary material

Figure S1

Figure S2

Figure S3

Figure S4

Figure S5

Figure S6

Figure S7

Figure S8

Figure S9

Figure S10

Data S1
