# Supplementary material for: Anti-mitotic chemotherapeutics promote apoptosis through TL1A-activated death receptor 3 in cancer cells
Source: Cell Res. 2018 Mar 1;28(5):544–55. doi: 10.1038/s41422-018-0018-6 (PMC5951888; doi:10.1038/s41422-018-0018-6)

Supplementary information, Figure S1. Susceptibility of a panel of cancer cell lines to diazonamide and taxol. Cells were treated with the indicated concentration of diazonamide (DA) or taxol (TX) for 48 hours.

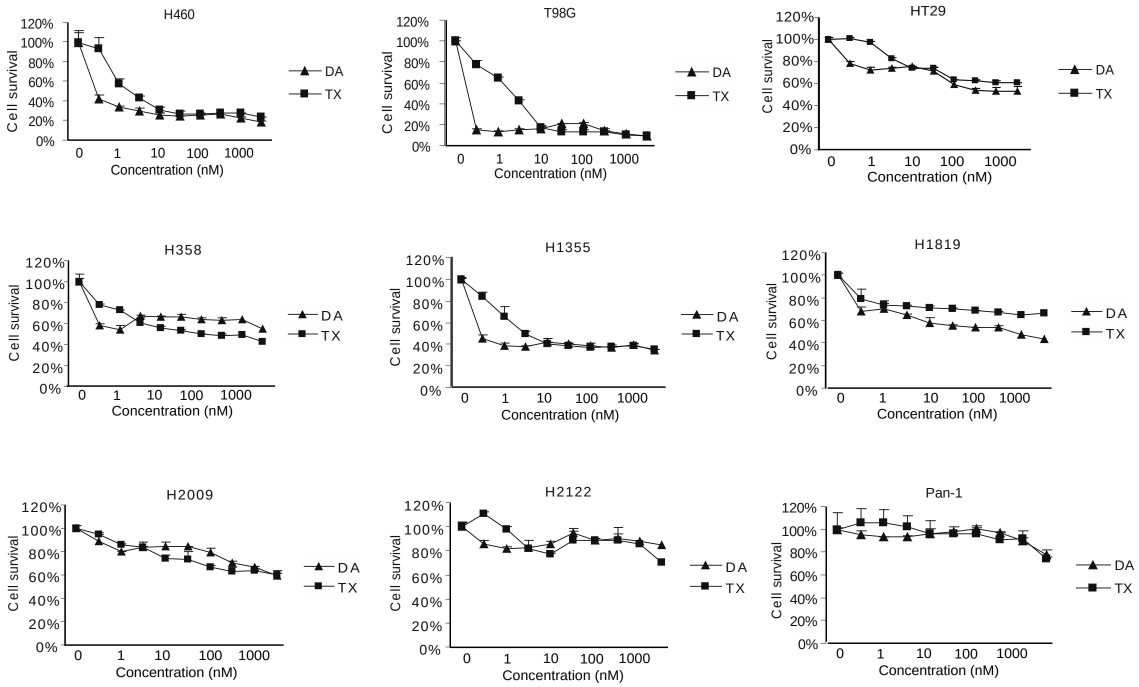

Supplement: Supplementary file 1 — Figure S1 [file 41422_2018_18_MOESM1_ESM.pdf]
