# Supplementary material for: Anti-mitotic chemotherapeutics promote apoptosis through TL1A-activated death receptor 3 in cancer cells
Source: Cell Res. 2018 Mar 1;28(5):544–55. doi: 10.1038/s41422-018-0018-6 (PMC5951888; doi:10.1038/s41422-018-0018-6)

Supplementary information, Figure S2. DR3 is required for anti-mitotics-induced apoptosis. HeLa cells stably expressing tetracycline (tet) – inducible DR3 shRNA were generated, designated as Hela-DR3shRNA. DR3-Flag with scrambled mutations in the shRNA targeting region was introduced into DR3shRNA cells to generate DR3shRNA/Scr cells. HeLa-DR3shRNA cells (A) or HeLa-SR3shRNA/Scr cells (B) were treated with or without tet for 48 h followed by the exposure to increasing concentrations of diazonamide. Cell viability was measured after 48 h of treatment. (C) DR3 mRNA levels in DR3 shRNA cells with or without tetracyclin induction. (D) In the HeLa cells stably expressing tetracycline (tet) inducible-DR3 shRNA, DR3-Flag protein was sustained in DR3shRNA/Scr cells even when DR3shRNA was expressed. Cell viability was measured after stable DR3 shRNA or DR3shRNA/Scr cells were treated with 100nM taxol (E) or 10nM vinblastine (F) for 48 h in the absence and presence of tetracycline. Values are presented as means ± SD (**p<0.01, ***p<0.001, NS: not significant). (G) DR3 shRNA cells were treated with 500nM diazonamide, vinblastine or taxol for 4 h in the absence or presence of tetracycline. Intracellular concentrations of the drugs were determined by LC-MS analysis. (H-I) DR3 shRNA or DR3shRNA/Scr cells were grown in the absence or presence of tetracyclin in the 24-well plates. Cell numbers were counted at the indicated time.

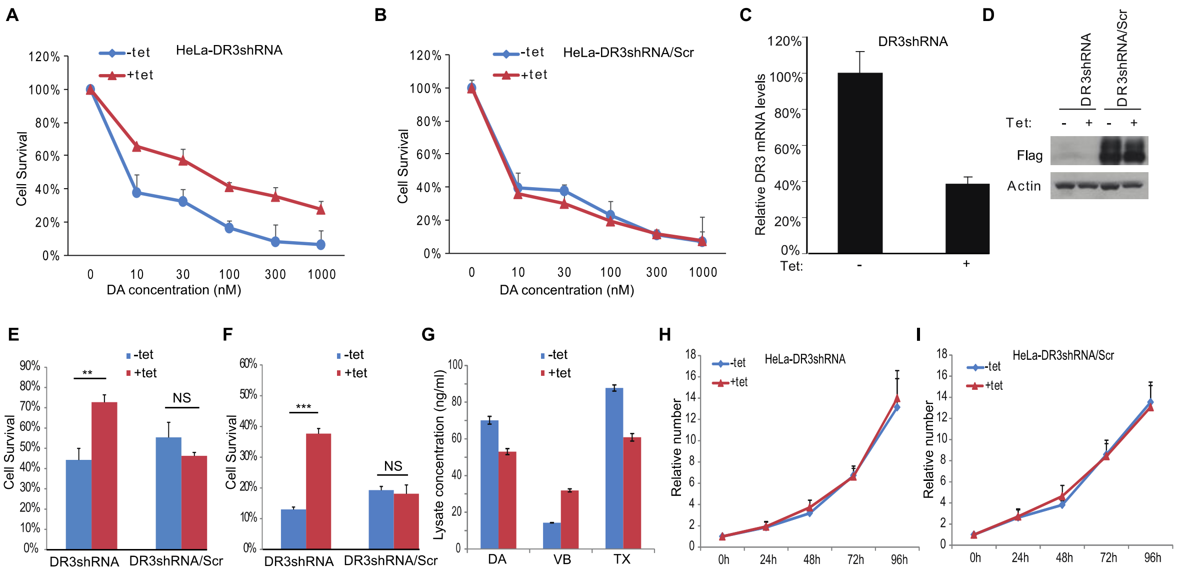

Supplement: Supplementary file 2 — Figure S2 [file 41422_2018_18_MOESM2_ESM.pdf]
