# Supplementary material for: Anti-mitotic chemotherapeutics promote apoptosis through TL1A-activated death receptor 3 in cancer cells
Source: Cell Res. 2018 Mar 1;28(5):544–55. doi: 10.1038/s41422-018-0018-6 (PMC5951888; doi:10.1038/s41422-018-0018-6)

Supplementary information, Figure S3. DR3 is required for diazonamide-induced cell death. (A) HeLa cells were transfected with siRNA for the indicated death receptors. After 48h of transfection, the cells were treated with 100nM diazonamide and the cell viability assay was performed after 48h of treatment. (B) The DR3 mRNA levels in knockdown cells were analyzed using quantitative RT-PCR. (C-E) Knockdown efficiency of siRNA oligos against TNFR1, Fas, DR4 or DR5 was reflected by functional assays. HeLa cells were transfected with siRNAs for TNFR1 (C), Fas (D), DR4 or DR5 (E). After 48 h, the cells were treated with 20 ng/ml TNF-α (C), 50 ng/ml FasL (D), or 100 ng/ml TRAIL (E) plus 1μM Smac mimetic for 48 h. (F) DR3 was not important in cell death induced by a DNA damage agent, doxorubicin. HeLa cells were transfected with DR3 siRNA for 48 h prior to treatment with 0.5μM doxorubicin or not for 48 h.

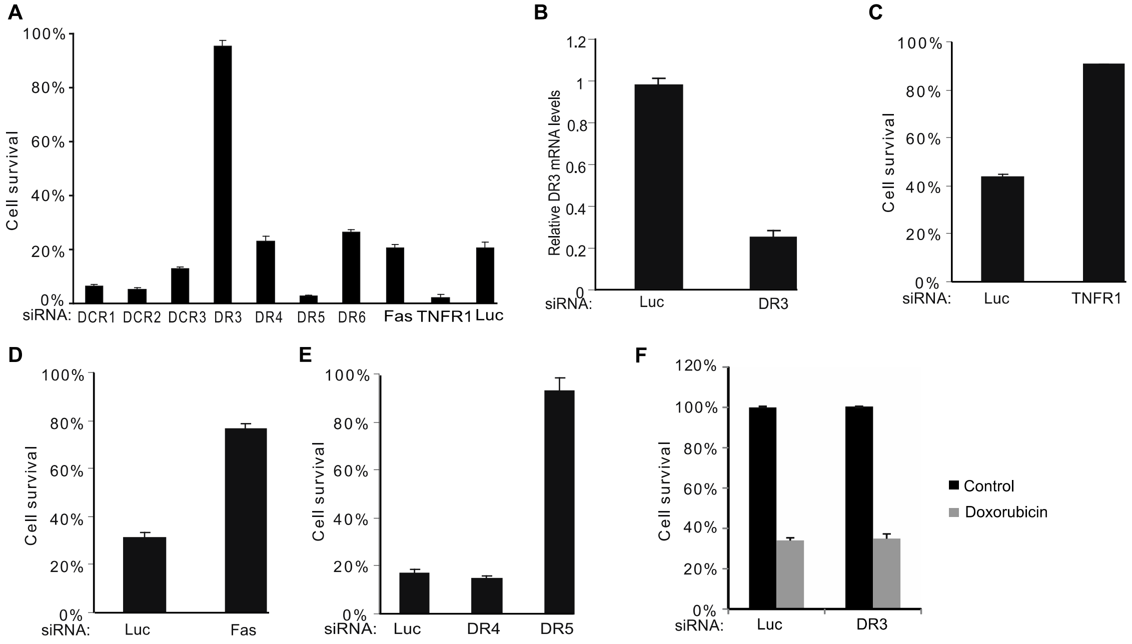

Supplement: Supplementary file 3 — Figure S3 [file 41422_2018_18_MOESM3_ESM.pdf]
