# Supplementary material for: Anti-mitotic chemotherapeutics promote apoptosis through TL1A-activated death receptor 3 in cancer cells
Source: Cell Res. 2018 Mar 1;28(5):544–55. doi: 10.1038/s41422-018-0018-6 (PMC5951888; doi:10.1038/s41422-018-0018-6)

Supplementary information, Figure S4. Exogenous expression of DR3 renders HT29 cells sensitive to anti-mitotics. (A) The dose response curves of HT29 cells to diazonamide and taxol. (B) HT29 and HT29-DR3 cells were seeded in the 24-well plates. Cell numbers were counted at the indicated time. (C-D) Dose response curves of taxol (C) or vinblastine (D) were plotted for HT29 cells and HT29-DR3 cells. (E) HT29-DR3 cells were transfected with multiple siRNA oligos targeting DR3. Cell viability was determined after 48 h treatment of 1nM vinblastine, 30nM diazonamide or taxol (lower panel). The knockdown efficiency of the DR3 siRNA oligos was analyzed by western blotting of the cell lysates with anti-Flag antibody (upper panel). (F) HT29 cells (top) and HT29-DR3 cells (bottom) were treated with DMSO or 50 nM taxol for 16 h, and collected for cell cycle profile analysis by flow cytometry.

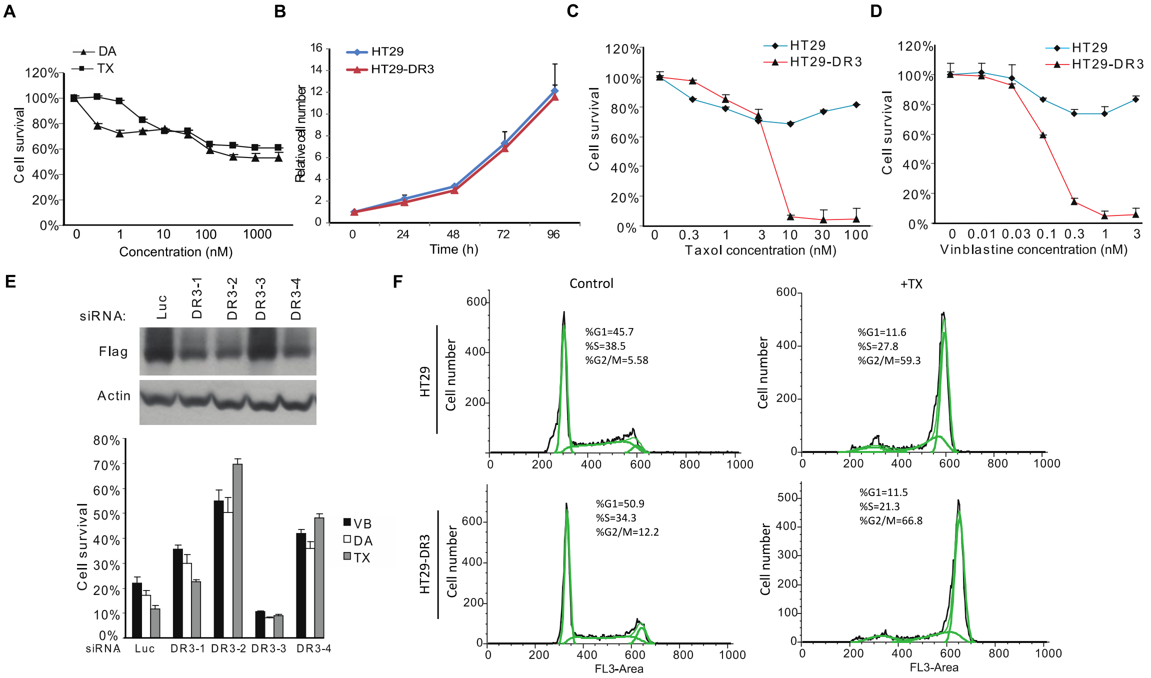

Supplement: Supplementary file 4 — Figure S4 [file 41422_2018_18_MOESM4_ESM.pdf]
