# Supplementary material for: Anti-mitotic chemotherapeutics promote apoptosis through TL1A-activated death receptor 3 in cancer cells
Source: Cell Res. 2018 Mar 1;28(5):544–55. doi: 10.1038/s41422-018-0018-6 (PMC5951888; doi:10.1038/s41422-018-0018-6)

Supplementary information, Figure S5. The responsiveness of HT29 cells and HT29-DR3 cells to a variety of drugs. HT29 cells and HT29-DR3 cells were treated with the indicated concentrations of six toxins for 48h, and the dose response curves of cell survival were generated.

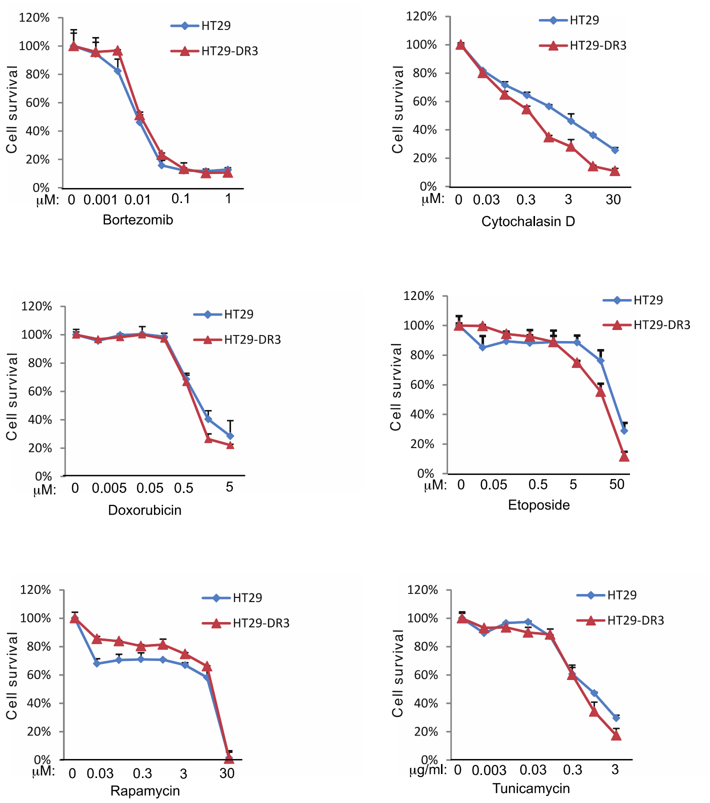

Supplement: Supplementary file 5 — Figure S5 [file 41422_2018_18_MOESM5_ESM.pdf]
