# Supplementary material for: Anti-mitotic chemotherapeutics promote apoptosis through TL1A-activated death receptor 3 in cancer cells
Source: Cell Res. 2018 Mar 1;28(5):544–55. doi: 10.1038/s41422-018-0018-6 (PMC5951888; doi:10.1038/s41422-018-0018-6)

Supplementary information, Figure S6. The formation of DR3/TL1A receptor complex and DISC complex. (A) HT29-DR3 cells were treated with 100nM taxol, 30nM vinblastine for 12 h. The DR3/TL1A complex was analyzed by Flag immunoprecipitation followed by western blotting with antibodies against Flag, TL1A, and TRADD. (B) HT29-DR3 cells were treated as in (A). The DISC complex was analyzed by caspase-8 immunoprecipitation followed by western blotting with antibodies against caspase-8 and FADD.

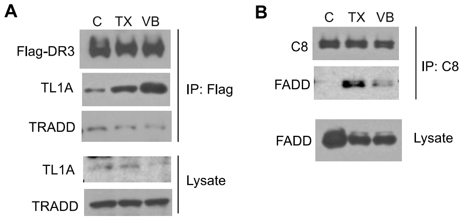

Supplement: Supplementary file 6 — Figure S6 [file 41422_2018_18_MOESM6_ESM.pdf]
