# Supplementary material for: Anti-mitotic chemotherapeutics promote apoptosis through TL1A-activated death receptor 3 in cancer cells
Source: Cell Res. 2018 Mar 1;28(5):544–55. doi: 10.1038/s41422-018-0018-6 (PMC5951888; doi:10.1038/s41422-018-0018-6)

Supplementary information, Figure S7. TL1A is important for apoptosis induced by taxol, diazonamide or vinblastine in a variety of cancer cell lines. Three-round transfection was performed with Luciferase or TL1A siRNA oligo followed by treatment with 100nM diazonamide (DA) or taxol (TX), or 30nM vinblastine (VB) for 48 h. Cell viability (left panel), TL1A mRNA levels (middle panel), and TL1A protein levels (right panel) were determined. The following cancer cells were tested: (A) T98G (brain glioblastoma), (B) MCF7 (breast cancer), (C) A549 (lung cancer), (D) HCC15 (lung cancer), (E) HeLa (cervical cancer), and (F) H1355 (lung cancer).

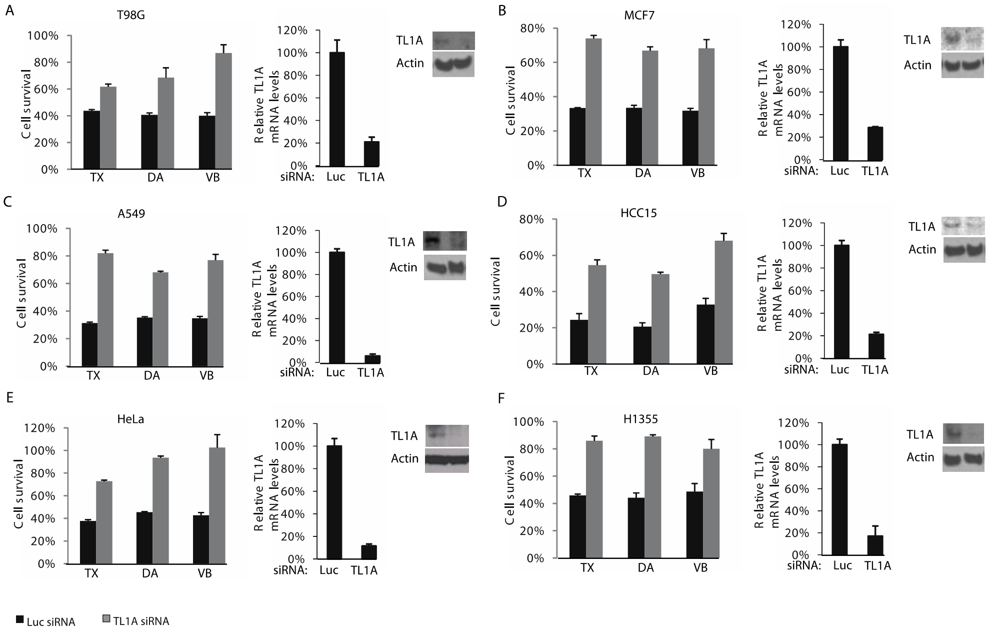

Supplement: Supplementary file 7 — Figure S7 [file 41422_2018_18_MOESM7_ESM.pdf]
