# Supplementary material for: Anti-mitotic chemotherapeutics promote apoptosis through TL1A-activated death receptor 3 in cancer cells
Source: Cell Res. 2018 Mar 1;28(5):544–55. doi: 10.1038/s41422-018-0018-6 (PMC5951888; doi:10.1038/s41422-018-0018-6)

Supplementary information, Figure S8. (A) The dose response curves of PANC-1 cells to diazonamide and taxol. (B-C) The dose response curves of PANC-1 cells and PANC-1-TL1A cells to doxorubicin and bortezomib. (D) HeLa cells were pretreated with z-VAD for 2 h followed by administration of 100nM taxol or 30nM vinblastine. After 16 h treatment, the conditioned cell culture media were removed for soluble TL1A analysis. (E) HeLa cells were treated with or without 100nM diazonamide for 16 h. TL1A levels in the culture medium were determined. Values are presented as means ± SD (***p<0.001). (F-H) HeLa cells were treated with the indicated concentrations of taxol (F), diazonamide (G), or vinblastine (H) in the presence or absence of TL1A (100ng/ml) for 48h. Cell viability was measured using Cell Titer Glo kit.

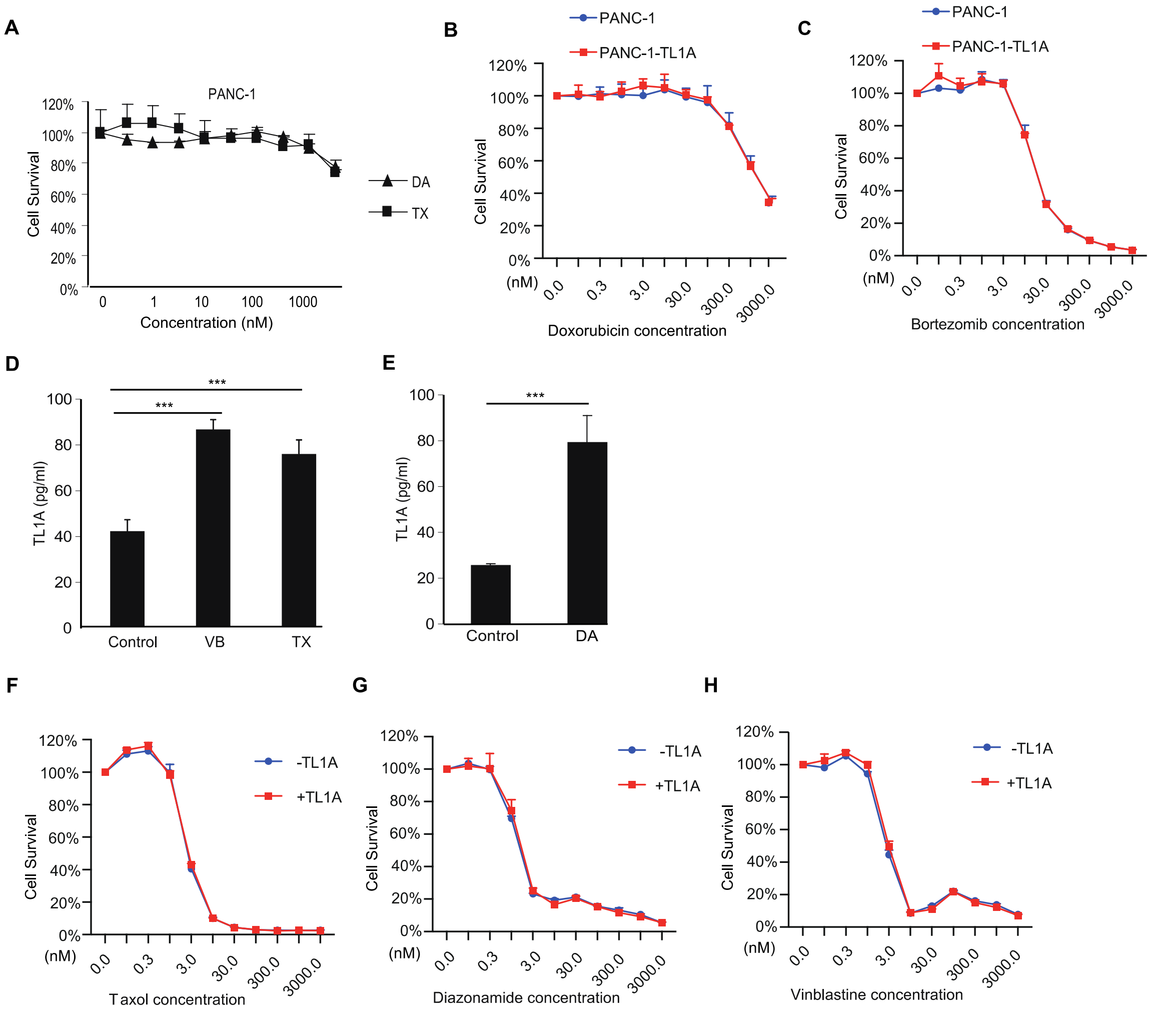

Supplement: Supplementary file 8 — Figure S8 [file 41422_2018_18_MOESM8_ESM.pdf]
