# Supplementary material for: Anti-mitotic chemotherapeutics promote apoptosis through TL1A-activated death receptor 3 in cancer cells
Source: Cell Res. 2018 Mar 1;28(5):544–55. doi: 10.1038/s41422-018-0018-6 (PMC5951888; doi:10.1038/s41422-018-0018-6)

Supplementary information, Figure S9. HT29 cells stably expressing DR3-Flag were incubated with 100nM taxol for the indicated time and stained with Flag antibody and DAPI. Scale bar: 10μm.

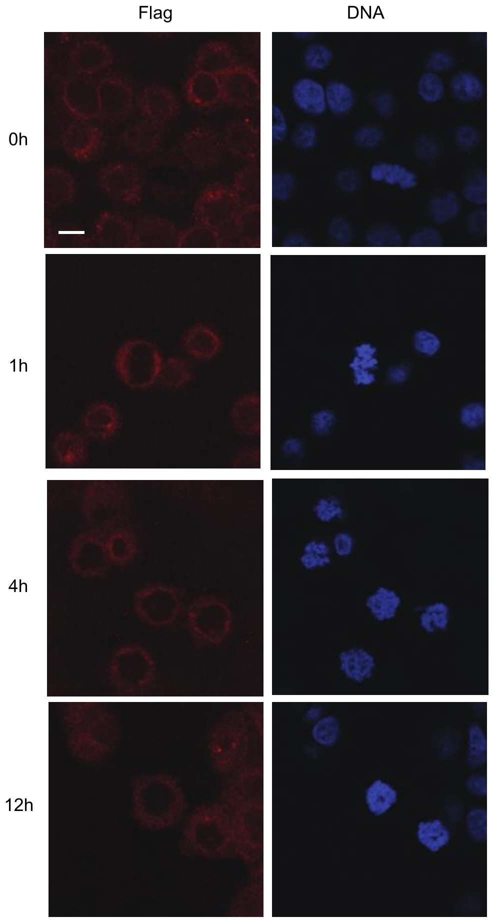

Supplement: Supplementary file 9 — Figure S9 [file 41422_2018_18_MOESM9_ESM.pdf]
