# Supplementary material for: Anti-mitotic chemotherapeutics promote apoptosis through TL1A-activated death receptor 3 in cancer cells
Source: Cell Res. 2018 Mar 1;28(5):544–55. doi: 10.1038/s41422-018-0018-6 (PMC5951888; doi:10.1038/s41422-018-0018-6)

Supplementary information, Figure S10. The abundance of DR3 and TL1A in tumor and normal samples from a range of tissue types from The Cancer Genome Atlas (TCGA).

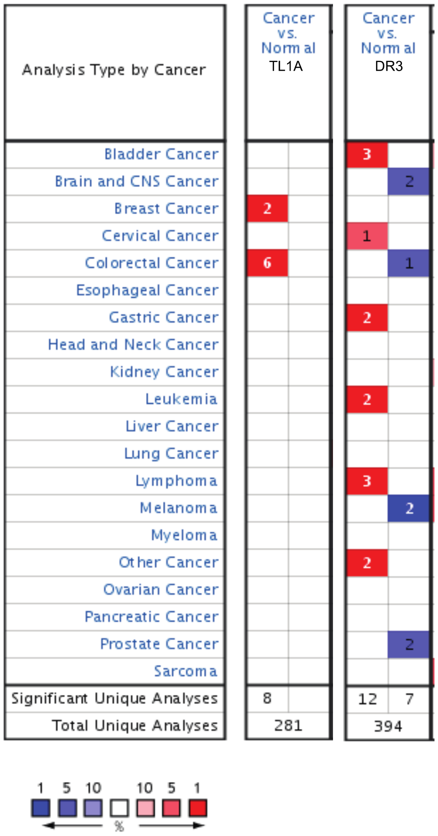

Supplement: Supplementary file 10 — Figure S10 [file 41422_2018_18_MOESM10_ESM.pdf]
