# Supplementary material for: Anti-mitotic chemotherapeutics promote apoptosis through TL1A-activated death receptor 3 in cancer cells
Source: Cell Res. 2018 Mar 1;28(5):544–55. doi: 10.1038/s41422-018-0018-6 (PMC5951888; doi:10.1038/s41422-018-0018-6)
Supplement: Supplementary file 11 — Data S1 [file 41422_2018_18_MOESM11_ESM.pdf]

Supplementary information, Data S1

Extended Experimental Procedures

Genome-wide siRNA screen for genes responsible for antimitotic-induced cell death

The screen was performed against the Dharmacon siRNA library, which contains pools of siRNA against 21,127 human genes. HeLa Tet-On cells grown in 96-well plates were transfected with siRNA in triplicates for 24 h following the treatment of 100nM diazonamide for another 48 h. The cell viability was determined using the CellTiter-Glo luminescent assay kit. Mad2 siRNA was used as positive control.

siRNA oligos

All siRNAs were obtained from Dharmacon. The pooled siRNA from siGENOME SMARTpool were for: TNFR1, DR3, DR4, DR5, DR6, Fas. The individual oligos were for:
Caspase-8-1 target sequence: 5-UGAAGAUAAUCAACGACUAUU-3;
Caspase-8-2 target sequence: 5-UGGAUUUGCUGAUUACCUAUU-3;
Caspase-8-3, obtained from Dharmacon [J-003466-14].
Caspase-9-1 target sequence: 5-GAUGCCUGGUUGCUUUAAUUU;
Caspase-9-2 obtained from Dharmacon [J-003309-05];
Caspase-9-3 obtained from Dharmacon [J-003309-06].
DR3-1 target sequence: GGACCAGUUGCCCAGCAGA;
DR3-2 target sequence: AGAAGAUCUGCACCGUCCA;
DR3-3 target sequence: CAAGAAGAUUGGUCUGUUU;
DR3-4 target sequence: GGAAGGAGUUCGUGCGCAC.
TL1A-1 target sequence: CGAAUGAACUAUACCAACA;
TL1A-2 target sequence: GACCAAGUCUGUAUGCGAA;
TL1A-3 target sequence: GGGACAAGCUAAUGGUGAA;
TL1A-4 target sequence: GGGCACACCUGACAGUUGU.

Quantitative RT-PCR analysis

Total RNA was extracted with RNA-BEE STAT-60 reagent following manufacture’s protocol. cDNAs were synthesized was with oligo(dT) primer using SuperScript II Reverse Transcriptase (Invitrogen). PCR was performed on an AB 7900HT fast real-time PCR system (Applied Bioscience) using DR3 or TL1A specific primer. Primer pair for DR3 is AGGTCAGCCAATGTGTCAG (forward) and AGCCATCGCCATGTTCATAG (reverse). Primer pairs for TL1A are CACCTCTTAGAGCAGACGGAGATAA (forward) and

TTAAAGTGCTGTGTGGGAGTTTGT (reverse); AGCAGGCCGACCAAACAA (forward) and GCTGTCTGTTACCTTGGTGATGAC (reverse).

Measurement of cell uptake of taxol, diazonamide and vinblastine

HeLa-DR3shRNA cells were grown in the absence and presence of tetracycline for 5 days before split into 10 cm plates. The following day, duplicate plates of each condition (+/- tetracycline) were incubated with diazonamide, vinblastine or Paclitaxel (500nM). Cells were harvested after 4 hours and resuspended in PBS at $1x10^6$ cells/ml. Aliquot of 250 μL of sample and blank were run in a speed-vac for approximately 1.5 hours to remove aqueous component. The cell pellets were resuspended in a mixture (1:1) of dH_2O: methanol crash solution containing 0.1 ng/μl n-benzylbenzamide at a volume of 175 μl. 160 μl samples were cleared and transferred to LCMS vials with inserts and analyzed by LCMS (4000 instrument). Compound Transitions are diazonamide: 697.3 to 498.2; vinblastine: 811.4 to 355.2; paclitaxel: 854.2 to 105.1.

Measurement of endotoxin

Endotoxin in recombinant TL1A was measured using ToxinSensor Chromogenic LAL kit (GenScript) following manufacturer's protocol.
